# Report of Miscellaneous Cases Occurring Since the Last Meeting of the Georgia Medical Society, Savannah, Ga.

**Published:** 1875-11

**Authors:** Henry LeHardy

**Affiliations:** Reporter


### REPORT OF MISCELLANEOUS CASES

Occurring Since the Last Meeting of the Georgia Medical Society, Savannah, Georgia.

HENRY LE HARDY, Reporter.

WEDNESDAY, August 25, 1875.

Dr. W. G. Bulloch, President, in the chair.

Dr. Geo. H. Stone reports that at 7 o'clock P.M. he was called to see a strong young negro woman, who had been attended in labor by a midwife. The child was born alive, and the woman had no hemorrhage. The woman was dead when Dr. Stone got to her house. She had been throwing her arms about and vomiting during the day.

Dr. J. C. Habersham, a case of tetanus: A boy of about 11 years of age, delicate, fell from a tree on the 31st of July and struck his head, inflicting a lacerated wound on the forehead. Stitches were applied to the wound, and cold water applications directed to be kept on the parts. Four days afterwards the stitches were removed, and the child began to complain of stiffness in his jaws; one side of his face was paralyzed, and his pupils were dilated. Difficulty in speaking and swallowing soon followed, but his mind has remained perfectly clear. He has been kept under the influence of hydrate of chloral, morphine, nitrite of amyle and bromide of potassium up to this time, but still suffers with some opisthotonos and difficulty in swallowing. The pulse in his case has never gone beyond 120, nor below 80. These symptoms seem to point out a combination of tetanus with some lesion of the brain. The case will probably terminate fatally.

Dr. J. G. Thomas: A negro woman in the fourth or fifth month of pregnancy had been delivered of twins on last Saturday morning. At 1 o'clock Dr. Easton Younge was called in to deliver the placenta, and removed what seemed to be a mass of rotten flesh. On Sunday Dr. Thomas took charge of the case, and found that the woman had a copious discharge of very fetid lochia. On Monday the discharge continued, and the patient showed some symptoms of blood poisoning. To-day she has peritonitis, and complains of a great deal of pain in the groin. The husband is decidedly syphilitic.

Dr. J. P. S. Houston: Was called out of town last Sunday at 3 o'clock A.M.; found a negro man with an incised wound of the abdomen, inflicted with a knife, about one inch above Poupart's ligament. The intestines protruded, and were found to be wounded. The wound in the walls of the abdomen being small, it had to be enlarged to allow the intestines to be replaced. Bandages were applied, and morphine prescribed. On Monday morning the pulse rose to 140, he was bathed in a cold perspiration and was vomiting at times, but felt otherwise comfortable until he died, at about 12 o'clock.

---

WEDNESDAY, September 1, 1875.

Dr. Juriah Harriss reports one case of puerperal convulsions preceding labor. When he was called to see the patient found her complaining of a very severe headache. On Monday morning she had a violent convulsion, and another one in the afternoon. The headache was still severe, but there was no œdema, the urine was very albuminous. At 3 o'clock this morning she was suffering from pains in the back, and labor commenced. She was in the seventh month of pregnancy.

Dr. Robert P. Myers: A case of intermittent fever occuring in an old negro man, and yielding readily to sulphate of cinchona.

Dr. Richard H. Lewis: A case of xerophthalmia in a negro boy æt. four years—principally interesting on account of its apparent cause. When first seen, the eyes had been affected for about a fortnight, and had not been treated at all. The conjunctivæ were dry and cuticular in appearance, wrinkling around the corners on movements of the ball. Large superficial phagedenic ulcer of the right cornea, with shrinkage of that globe to two-thirds of its natural size, and atrophy of the left optic nerve. The mother states that, for a week, the child had shed no tears when it cried; that it was rapidly loosing flesh; and, if allowed, would sleep all the time. He was exceedingly stupid, and evidently suffering from brain trouble, possibly tubercular meningitis. As the child had had no chronic conjuctivitis, nor had been the victim of violent caustics, the usual causes of xerophthalmia, Dr. Lewis attributed the atrophy of the secretory apparatus of the conjunctiva with the other atrophic changes to the brain disease. Ordered oil and atropine as a lubricator and soothing agent, but gave a gloomy prognosis, and advised the mother to consult a general practitioner for the treatment of the more dangerous affection.

Drs. J. C. Habersham and R. D. Arnold: In the case of tetanus reported last week the temperature has become normal, but the spasms still continue. The use of morphine by hypodermic injections had to be discontinued, the introduction of the needle producing spasms. As the paroxysms occur principally every other night, quinine has been administered as an antiperiodic and nervine, but without any benefit.

Dr. George H. Stone was called to see a white boy, but found him dead when he reached the house. He had treated the boy for sometime for fever. The parents stated that the boy had been sitting on a chair, when they noticed he had suddenly dropped asleep; they picked him up from the chair and put him on a bed, where he died in a few minutes.

Dr. Theo. Starbuck delivered a lady of a child on the 20th of August. A few days ago he was called to see her for an eruption she had on the vulva. The child had the same eruption on the scrotum, and the nurse and two other female attendants have contracted the same eruption on their hands. This eruption consists of small pimples, not more than one-sixth of an inch in diameter, with an exudation of serum from the apex. This exudation dries up, scabs off, and leaves the parts raw and sore.

Dr. J. G. Thomas: On last Friday I saw in confinement a negro girl fifteen years old. The girl was unconscious, and very nervous and delirious; womb dilating. After a time found the womb still dilating, and ruptured the membranes. I came in again later and found the head tightly wedged in the pelvis. I applied the forceps and delivered the mother of a dead child.

The mother remained unconscious during the whole of the labor. She had a very quick pulse, and remained in the same nervous, unconscious state until she died, last Monday morning.

On Sunday morning saw a woman in natural labor, and delivered her of a healthy boy. The placenta was delivered with not more than the usual flow of blood. Dr. Thomas was waiting to apply the bandage until the nurse had finished dressing the child, when he noticed that the woman's face was turning perfectly white, and she soon fell in a deep syncope, but after the administration of stimulants she soon recovered. Yesterday the temperature rose, and she was having a little fever. The heart's impulse is weak, and the woman is known to faint from very very slight causes.

